# Bacteriophage-mediated approaches for biofilm control

**DOI:** 10.3389/fcimb.2024.1428637

**Published:** 2024-10-07

**Authors:** Arianna Mayorga-Ramos, Saskya E. Carrera-Pacheco, Carlos Barba-Ostria, Linda P. Guamán

**Affiliations:** ^1^ Universidad UTE, Centro de Investigación Biomédica (CENBIO), Facultad de Ciencias de la Salud Eugenio Espejo, Quito, Ecuador; ^2^ Escuela de Medicina, Colegio de Ciencias de la Salud Quito, Universidad San Francisco de Quito USFQ, Quito, Ecuador; ^3^ Instituto de Microbiología, Universidad San Francisco de Quito USFQ, Quito, Ecuador

**Keywords:** bacteriophages, biofilms, antimicrobial design, antibiotic resistance, exopolysaccharide

## Abstract

Biofilms are complex microbial communities in which planktonic and dormant bacteria are enveloped in extracellular polymeric substances (EPS) such as exopolysaccharides, proteins, lipids, and DNA. These multicellular structures present resistance to conventional antimicrobial treatments, including antibiotics. The formation of biofilms raises considerable concern in healthcare settings, biofilms can exacerbate infections in patients and compromise the integrity of medical devices employed during treatment. Similarly, certain bacterial species contribute to bulking, foaming, and biofilm development in water environments such as wastewater treatment plants, water reservoirs, and aquaculture facilities. Additionally, food production facilities provide ideal conditions for establishing bacterial biofilms, which can serve as reservoirs for foodborne pathogens. Efforts to combat antibiotic resistance involve exploring various strategies, including bacteriophage therapy. Research has been conducted on the effects of phages and their individual proteins to assess their potential for biofilm removal. However, challenges persist, prompting the examination of refined approaches such as drug-phage combination therapies, phage cocktails, and genetically modified phages for clinical applications. This review aims to highlight the progress regarding bacteriophage-based approaches for biofilm eradication in different settings.

## 1 Introduction

Biofilms are sophisticated microbial communities aggregated in a self-generated extracellular matrix, which anchors the cells together and facilitates communication and resource distribution among them (Penesyan et al., 2021). The architecture of biofilms endows them with distinct characteristics compared to planktonic (free-living) cells, including altered metabolism and enhanced resistance to external stressors (Lee et al., 2014; Yin et al., 2019).

The clinical significance of biofilms predominantly stems from their heightened resistance to antimicrobial agents, posing an enormous challenge in treating biofilm-associated infections (Schulze et al., 2021; Ali et al., 2023). Cells within biofilms exhibit antimicrobial resistance that can be up to a thousand times greater than that of planktonic cells (Mah and O’Toole, 2001; Sharma et al., 2019). Biofilms are implicated in a wide array of chronic infections and are responsible for the failure of numerous antimicrobial treatments, particularly in the context of medical device- and tissue-associated infections. The current treatment regimens, often relying on conventional antimicrobials, are increasingly proving inadequate in eradicating these resilient microbial communities (Usui et al., 2023).

Given the limitations of existing therapies and the escalating threat of multidrug-resistant microbes, there is an urgent need to develop novel anti-biofilm strategies. Innovative approaches, such as the use of antimicrobial peptides, nanotechnology, surface modifications of medical devices, and bacteriophage applications, are being explored to combat biofilm-associated infections (Yasir et al., 2018; Li et al., 2020; Mohanta et al., 2023; Kushwaha et al., 2024). Understanding the complex biology of biofilms and exploring bacteriophages as potential biocontrol agents are critical in addressing the biofilm-related challenges in medical and industrial environments. This review aims to provide a comprehensive overview of the basic biofilm biology, the resistance mechanisms within biofilms, and the emerging role of bacteriophages in biofilm control.

## 2 Biofilm formation

Biofilms, crucial in diverse environments, are complex microbial communities encapsulated in an extracellular polymeric substances (EPS) matrix (Li et al., 2022; Krishnan et al., 2023). Understanding each step of biofilm formation—from initial attachment through irreversible adhesion, microcolony formation, maturation, to dispersion—is crucial for developing effective eradication strategies. Each phase presents unique targets and mechanisms that can be disrupted to prevent biofilm formation or to dismantle existing biofilms. For instance, interventions at the initial attachment stage can prevent biofilm establishment, while strategies targeting biofilm maturation can disrupt the protective EPS matrix, enhancing the efficacy of antimicrobial agents. Therefore, detailed knowledge of these processes is essential for devising comprehensive and effective biofilm control measures, ultimately improving infection management and patient outcomes (Armbruster and Parsek, 2018; Nie et al., 2021).

1. Initial attachment: The initial phase involves microorganisms adhering to surfaces via physical forces (van der Waals forces, electrostatic attractions, hydrophobic effects). Bacterial appendages play a crucial role in surface movement, enabling the bacteria to explore and find optimal attachment sites. Additionally, pili, such as Type I and Type IV, facilitate adhesion by establishing strong interactions with the surface. These initial interactions are reversible, allowing the bacteria to detach and relocate if the conditions are unfavorable (Richter et al., 2017; Ramzan et al., 2022).
2. Irreversible attachment: The transition to irreversible attachment is marked by the production of EPS, which anchors the bacteria firmly to the surface. This EPS matrix not only strengthens adhesion but also forms a protective barrier that shields the microbial community from environmental stressors, including antimicrobial agents and components of the immune response. During this phase, these microorganisms can undergo significant genetic shifts, prioritizing the synthesis of EPS and reducing the expression of motility-related genes (Chávez-Jacobo et al., 2023; Guttenplan et al., 2010; Kim et al., 2023a).
3. Microcolony formation: As bacteria continue to produce EPS and proliferate, they form structured microcolonies, which are clusters of bacterial cells within the EPS matrix. This stage is characterized by spatial organization and differentiation within the biofilm, influenced by environmental gradients such as nutrient availability, oxygen concentration, and waste accumulation. Quorum sensing, a key cell-density signaling mechanism, is crucial in regulating gene expression associated with EPS production and biofilm maturation. This process ensures coordinated behavior among the bacterial population, facilitating efficient biofilm development (Zhang et al., 2018; Oyewole et al., 2022; Gulec and Eckford, 2023).
4. Biofilm maturation: In this phase, microcolonies merge, creating a layered, three-dimensional structure. EPS is crucial for structure and protection, creating diverse microenvironments within the biofilm. These niches allow for metabolic specialization among microbial populations. Additionally, the development of water channels, which occurs in this phase, is essential for nutrient distribution and waste removal, while quorum sensing ensures coordinated gene expression throughout the biofilm, regulating behaviors like virulence and resistance (Azulay et al., 2022; Xiu et al., 2022).
5. Dispersion: During this phase, cells detach from the mature biofilm to colonize new surfaces through passive mechanisms, such as fluid shear, and active, regulated processes initiated by environmental changes. Active dispersion involves the degradation of the EPS matrix, often facilitated by enzymes such as dispersin B, as well as phenotypic shifts that enable bacteria to revert to a planktonic, mobile phase (Rumbaugh and Sauer, 2020). Environmental triggers such as changes in nutrient levels or oxygen availability can initiate active dispersion, leading to the release of bacteria to colonize new environments. This process is crucial in clinical settings as it facilitates the spread of infections, particularly on medical devices, and contributes to the difficulty in treating biofilm-associated infections due to the rapid re-establishment of biofilms and increased resistance to antimicrobial agents (Werneburg et al., 2019).

Understanding these stages helps devise strategies to manage biofilm-related issues, emphasizing the dynamic nature of microbial communities and their interaction with environments. The study of biofilms bridges microbiology, molecular biology, and environmental sciences, offering insights into microbial behavior and potential control methods in medical and industrial contexts.

## 3 Bacteriophages-biofilm interaction in nature

The interactions between bacteriophages and biofilms are complex and rely on several factors determining biofilm destruction, coexistence, or limited phage efficacy in controlling biofilms.

The susceptibility of bacterial cells to phage infection can lead to the destruction of biofilms. When the biofilm is susceptible, bacteriophages attach to receptors on bacterial cells, inject their genetic material, and take over the bacterial machinery to replicate (Sutherland et al., 2004), leading to the lysis of infected cells and destroying the biofilm (Fernández et al., 2018; Papaianni et al., 2020). The availability of specific receptor sites on the biofilm bacteria’s surface is crucial for phage attachment and infection (Uchiyama et al., 2014). The polysaccharide-degrading enzymes produced by bacteriophages weaken the biofilm structure, allowing phages to penetrate and disperse the biofilm, releasing individual bacteria, making them more susceptible to other antimicrobial agents (González et al., 2018; Chegini et al., 2020). Phages can also produce endolysins, which hydrolyze the peptidoglycan in bacterial cell walls from within, contributing to the release of newly formed viral particles (Abdelrahman et al., 2021).

The biofilm composition and structure affect both the coexistence of phages with bacteria and limit the effectiveness of phages in controlling biofilm formation. The biofilm’s extracellular matrix, composed of polysaccharides, proteins, and DNA, can hinder phage diffusion and penetration, preventing them from reaching and killing biofilm bacteria (Limoli et al., 2015). In some cases, phages can promote biofilm formation and trigger specific responses in bacteria, increasing the adhesion, stability, and matrix production, allowing phages and bacteria to coexist within the biofilm (Secor et al., 2020). The presence, physiological state, and physical environment of different microorganisms (bacteria and fungi) influence phage accessibility to the biofilm (Bull et al., 2018; Ferriol-González and Domingo-Calap, 2020).

It is important to note that the interaction between phages and biofilms is still an active area of research. Further studies are needed to fully understand the complex dynamics between phages and biofilms in different environments and bacterial species.

### 3.1 Advantages and limitations of bacteriophages as antibacterial agents

Bacteriophages possess features such as self-replication within host bacteria, leading to increased concentrations in biofilms, and rapid replication, achieving high population densities swiftly (Talapko and Škrlec, 2020). They exhibit lytic activity against bacteria and produce enzymes that degrade the biofilm matrix, thereby enhancing biofilm penetration, infection, and elimination (Abedon, 2015). The continuous interactions between phages and hosts in biofilms drive evolutionary adaptations, potentially enhancing phage efficacy in disrupting biofilms (Talapko and Škrlec, 2020). All those characteristics, which are part of phages’ lifestyle are advantageous to use them as antibacterial agents.

On the other hand, limitations include the high specificity of bacteriophages for their hosts and the development of phage resistance by biofilm bacteria (Talapko and Škrlec, 2020). Bacterial defense mechanisms within the biofilm, such as CRISPR-Cas systems, plasmids, genomic islands, modification and mutation of surface receptors, or production of substances that inhibit phage attachment, prevent bacteriophages from infecting and eliminating biofilm communities (Cady and O’Toole, 2011; Römling and Balsalobre, 2012; Hassan et al., 2021; Ngiam et al., 2022). In addition, the biofilm matrix acts as a physical barrier, hindering phage movement and penetration (Talapko and Škrlec, 2020; Hassan et al., 2021; Ngiam et al., 2022).

## 4 Phage penetration and innovative phage delivery systems

### 4.1 Penetration strategies

Bacteriophages have several strategies for penetrating biofilms, one of which involves the production of an enzyme called polysaccharide depolymerase. This enzyme can digest polysaccharide components of bacterial cell walls by identifying and binding to specific ligands on the bacterial surface, facilitating phage genome transfer (Knecht et al., 2019). Depolymerases are subdivided into several classifications according to their target in biofilms: capsular polysaccharides, lipopolysaccharides (LPS), O-polysaccharides or exopolysaccharides (Pires et al., 2016; Knecht et al., 2019). Based on their biochemical properties, depolymerases are classified into hydrolases and lyases. Phage hydrolases catalyze the breaking of chemical bonds with water, displaying activities such as sialidases, xylosidases, levanases, rhamnosidases, dextranases, and peptidases. Conversely, lyases catalyze the breaking of chemical bonds through mechanisms other than hydrolysis, encompassing hyaluronate, alginate, and pectin/pectate lyases (Pires et al., 2016; Knecht et al., 2019; Topka-Bielecka et al., 2021). These depolymerases exhibit high substrate specificity, playing a crucial role in the bacteriophage’s ability to attach, invade, and ultimately destroy host bacteria (Yan et al., 2014).

Interestingly, bacteriophages have been genetically modified to express depolymerase during infection, enabling the simultaneous degradation of the bacterial cell wall and the biofilm matrix, thereby enhancing the chances of biofilm destruction (Lu and Collins, 2007). Furthermore, phages can produce endolysins, bactericidal proteins that hydrolyze bacterial cell walls, facilitating the release of phage particles (Briers et al., 2009; Abdelrahman et al., 2021). These endolysins, in conjunction with proteins such as holin, breach the bacterial cytoplasmic membrane to access the peptidoglycan layer (Wang et al., 2000). Additionally, the protein spanin contributes to the degradation of the outer membrane in Gram-negative hosts, further aiding in the bacteriophage’s infection process (Cahill and Young, 2019).

Phages can also switch between two life stages, lytic and lysogenic, contributing to their ability to penetrate and destroy the biofilm. During the lysogenic phase, the phage’s DNA merges with the bacterium’s genetic material, enabling the phage to penetrate and spread within the biofilm without destroying the host cells immediately (Weinbauer et al., 2003). This allows the phages to remain dormant until favorable conditions are present in the biofilm (Weinbauer et al., 2003). The control of the lytic and lysogenic phases involves various elements. Regulatory factors, including bacteriophage Lambda repressors CI and Cro, determine the lysogenic state; these elements were identified in previous studies (Carrasco et al., 2016; Lee et al., 2022). Other factors that can impact the lysogeny-lysis switch of phages are quorum sensing and the metabolic state of the host (Laganenka et al., 2019).

In clinical settings, an alternative to effectively reach and destroy biofilms is combining multiple bacteriophage mixtures with different host ranges into one solution. Bacteriophage cocktails can easily penetrate and destroy biofilms such as the ones from *Pseudomonas aeruginosa*, *Enterococcus faecalis*, and Methicillin-resistant *Staphylococcus aureus* (MRSA) (Hanlon, 2007; Kaur et al., 2016; Kwiatek et al., 2017; Khalifa et al., 2018). Another alternative is the phage-antibiotic synergy (PAS). In this approach, the phages can initiate biofilm breakdown, allowing antibiotics to penetrate more effectively and kill the bacteria more efficiently. Conversely, antibiotics can weaken the bacteria, making them more susceptible to phage infection (Morrisette et al., 2020). Other approaches involve using nanoparticles to improve phage penetration. For instance, a study by Quan et al. (2019) used magnetic nanoparticles to create artificial channels in infectious biofilms, improving the penetration and effectiveness of antimicrobial treatment. The experiment successfully demonstrated the creation of artificial channels in *S. aureus* biofilms, which significantly enhanced the penetration of the antibiotic gentamicin and the bacterial killing efficacy. This method offers a simple and effective way to eradicate infectious biofilms when combined with existing antibiotic therapies (Quan et al., 2019). The following sections provide more examples of these approaches.

Additionally, targeting ligands or antibodies can facilitate phages’ penetration through the biofilm matrix, effectively reaching and infecting bacterial cells (Amankwah et al., 2021). Phages can also be genetically engineered to produce cell wall-degrading enzymes and display biofilm-specific peptides, thus allowing further binding and penetration of biofilms (Chegini et al., 2020; Zhao et al., 2023). Notably, the accessibility of phages to the biofilm can be affected by the types of microorganisms forming the structure, their physiological state, and the physical environment.

### 4.2 Nanoparticle-based delivery

Nanoparticle-based delivery systems have emerged as a promising strategy to enhance the efficacy of bacteriophage therapy in biofilm control by using the unique properties of nanoparticles to improve phage stability, targeting, and penetration into biofilms (Vera-González and Shukla, 2020). By combining bacteriophages with nanoparticles, natural compounds, and disinfectants, the destruction of biofilms can be more effective (Chegini et al., 2020). Various types of nanoparticles have been used to improve phage stability, targeting, and penetration into biofilms, including metallic (e.g., silver and gold nanoparticles) and polymeric (e.g., Poly Lactic-co-Glycolic Acid (PLGA) and chitosan) nanoparticles (Ahiwale et al., 2017; Abdelsattar et al., 2021).

Chitosan nanoparticles have several outstanding properties that make them useful in various biomedical applications, such as biocompatibility and biodegradability (Ke et al., 2021). Additionally, they can encapsulate and release drugs in a controlled manner, improving the efficacy of pharmacological treatments and reducing side effects; for biofilm control and eradication, chitosan has been combined with bacteriophages (Sharifi-Rad et al., 2021). Encapsulating bacteriophages in chitosan nanoparticles can protect them from harsh gastrointestinal conditions and improve delivery to the target site for effective results. Adamu and coworkers synthesized chitosan nanoparticles to encapsulate phages targeting *Escherichia coli*, offering considerable protection of the ΦKAZ14 bacteriophage against enzymatic degradation and acidic environments. This innovation makes the phage suitable for oral applications, improving its delivery (Adamu Ahmad et al., 2016). In a similar approach using chitosan, Li and coworkers introduced an innovative method of using polyvalent phages attached to magnetic colloidal nanoparticle clusters (CNCs) under a small magnetic field. The phage PEL1 immobilized onto chitosan-coated Fe_3_O_4_-based CNCs showed enhanced phage loading and improved biofilm penetration. The author concluded this conjugation method could broaden the use of phages for microbial control by improving their delivery to relatively hard-to-reach areas within biofilms (Li et al., 2017).

Gold nanoparticles (AuNPs) have been used to target phages in *P. aeruginosa* biofilms, demonstrating a significant reduction in biofilm production, which suggests that phage-inspired AuNPs could serve as potent therapeutic agents against human pathogens (Ahiwale et al., 2017). Similar research used AuNPs but combined with the C3 phage to treat *P. aeruginosa* in planktonic and biofilm states. This combination exhibited high stability under various temperatures, pH levels, and salt concentrations, indicating a more efficient delivery (Abdelsattar et al., 2021).

In a recent approach, a phage-Chlorin e6 (Ce6)-manganese dioxide nanocomposite (PCM) was created, combining the benefits of phage therapy with other therapeutic modalities. The phage component of the nanocomposite plays a crucial role in targeting host bacteria and aiding in the efficient delivery of Ce6 to penetrate biofilms. By incorporating phages into the nanocomposite, the study improves the targeting of host bacteria, facilitating the effective delivery of Ce6 within the biofilm structure (Wang et al., 2023).

Similarly, Manoharadas and coworkers showed an enhanced phage delivery method by combining green-synthesized silver nanoparticles and bacteriophages to effectively disperse pre-formed *S. aureus* biofilms from inert surfaces. This approach not only removes the biofilms but also prevents the establishment of new infections and subsequent colonization by causing the loss of viability of the biofilm-entrapped bacterial cells (Manoharadas et al., 2021).

Recent advancements in nanoparticle-based delivery systems have emphasized the importance of surface modifications and functionalization to enhance their further interaction with biofilms and bacteriophages. The attachment of polyethylene glycol (PEG) chains to nanoparticles, known as PEGylation, has improved nanoparticles’ stability and circulation time, thereby enhancing the delivery of bacteriophages to biofilm sites (Mura et al., 2013). Additionally, dual-functional nanoparticles that combine targeting ligands with antimicrobial agents offer a promising strategy for enhancing the specificity and efficacy of bacteriophage therapy. For example, mannose-functionalized nanoparticles have been developed to target lectin receptors on the surface of biofilm-forming bacteria, improving phage adherence and biofilm disruption (Pawde et al., 2020). Furthermore, stimuli-responsive nanoparticles that release their payload in response to environmental triggers such as pH or enzymatic activity have shown potential in improving the targeted delivery of bacteriophages within biofilms (Karimi et al., 2016).

### 4.3 Liposome and hydrogel encapsulation-based delivery

Liposomes and vesicles constitute a versatile delivery system for hydrophilic and hydrophobic substances and represent the most promising vehicles for a variety of therapeutic agents, including bacteriophages. Liposome and vesicle encapsulation have recently emerged as an efficient approach for enhancing the efficient delivery and improved the efficacy of phages used to treat biofilms (Kaszuba et al., 1997; Sihorkar and Vyas, 2001). These exploit lipid-based carriers’ biocompatibility and tailor-made nature to improve biofilm phage interactions such as stability, targeting, or penetration into the biofilm structures (Singla et al., 2016).

The mechanisms to enhance bacteriophage therapy by using liposomes are: (I) Protection from environmental stress, where the encapsulation of bacteriophages in liposomes protects them from different environmental stressors, such as pH changes and enzymatic degradation, and thereby maintain their infectivity (Loh et al., 2021), (II) improved penetration into the biofilm thanks to the presence of the lipid bilayer structure that facilitates the delivery of phages into biofilms due to easy fusion with the bacterial membrane (Malik et al., 2017), and (III) targeted delivery through the functionalization with the targeting moieties to ensure that the phages are specifically targeted on the bacterial biofilms, which decreases off-target effects and enhances the therapeutic impact.

Recent advancements in liposomes and hydrogels for bacteriophage delivery have demonstrated significant potential in enhancing biofilm removal. In the context of orthopedic device infections, engineered injectable hydrogels encapsulating *P.aeruginosa* bacteriophages have shown promising results. These hydrogels, capable of controlled phage release, retain bacteriolytic activity and effectively target planktonic and biofilm bacteria without affecting human mesenchymal stromal cells. The authors concluded that the hydrogels efficiently deliver bacteriophages to treat localized bone infections (Wroe et al., 2020).

Furthermore, incorporating bacteriophages into liposomes and other amphiphilic nanoparticles offers the advantage of targeted delivery through ligand interaction with target cells. Although this approach presents challenges, such as ensuring amphiphilic properties or conjugation with anchoring molecules, it provides a sophisticated means of enhancing the directed delivery of bacteriophages (Loh et al., 2021).

Finally, an innovative delivery system has been developed for local tissue regeneration and infection control using bacteriophage-loaded alginate-nanohydroxyapatite hydrogel. This system efficiently encapsulates bacteriophages, with release influenced by environmental pH, without compromising their viability or functionality, thus improving their delivery. The hydrogels showed good tissue response and exhibited excellent antimicrobial effectiveness, inhibiting the attachment and colonization of multidrug-resistant *E. faecalis* (Barros et al., 2020).

Together, these studies illustrate the diverse strategies and significant progress made in using nanoparticle-encapsulated bacteriophages to address biofilm-related infections, paving the way for innovative treatments that enhance targeted delivery and efficacy.

### 4.4 Genetically engineered bacteriophages

**Figure 1:**
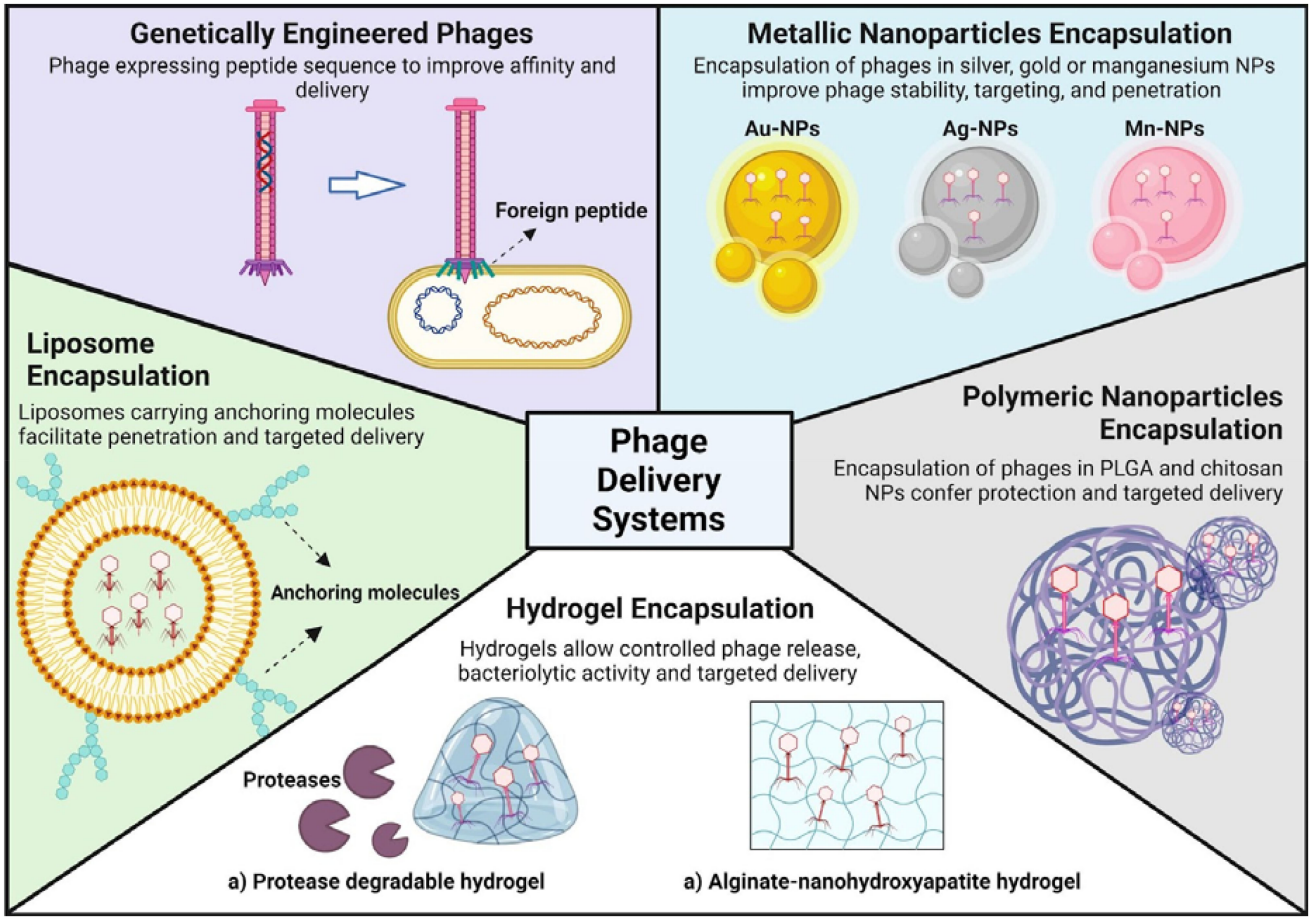
Advanced encapsulation strategies for phage delivery systems. The figure illustrates various methodologies for enhancing the stability and targeting of phages for therapeutic applications. Genetically engineered phages are modified to express specific peptides that improve their delivery efficiency. Liposomes, metallic nanoparticles (silver, gold, magnesium, etc.) and polymeric nanoparticles (PLGA and chitosan) are utilized to encapsulate phages, thereby bolstering their stability, penetration capabilities and ensuring targeted delivery. Moreover, the use of hydrogels (protease-degradable and alginate-nanohydroxyapatite) allows for controlled release and targeted delivery while maintain the phages innate bacteriolytic activity.

Phage engineering has shown promise in enhancing the effectiveness of tailored phage therapies in combating biofilm-related challenges. Most genetic engineering approaches for biofilm control focus on improving the specificity, affinity, and efficacy of bacteriophages rather than enhancing their delivery to eradicate biofilms (Lu and Collins, 2007; Gutiérrez et al., 2016; Chegini et al., 2020; Yue et al., 2022). Another application of genetic engineering of phages is the potential to extend their natural host range and modify phage display (Khambhati et al., 2023). However, improved delivery is often a beneficial consequence rather than the primary goal of these modifications. Recently, to overcome the challenge of eradicating biofilms in water distribution systems, the filamentous coliphage M13 was engineered to enhance biofilm affinity and deliver lytic polyvalent phages. Modified M13 showed a significantly higher affinity for *P.aeruginosa* biofilms and improved delivery capabilities (Sun et al., 2022). This tunable approach could enable enhanced phage delivery and higher biofilm eradication efficacy to expand the scope of phage applications. A summary of the innovations regarding phage delivery is shown in **Figure 1**.

Exploring innovative approaches to intentionally enhance phage delivery, such as engineering phages to improve phage entrance or withstand harsh conditions within biofilms, could offer significant advancements in biofilm control strategies. This gap suggests an opportunity for innovative approaches to enhance phage delivery to biofilms. For example, engineering phages to produce surface modifications that increase their ability to penetrate biofilms or survive harsh biofilm environments could significantly improve their therapeutic efficacy. Such targeted delivery strategies would be a novel direction for research and development in bacteriophage therapy.

## 5 Using bacteriophages to dismantle biofilms

The rise of antibiotic-resistant microorganisms has become a significant global public health concern, especially in chronic infections associated with bacterial biofilms growing over medical instruments. These biofilms demonstrate heightened antibiotic tolerance and host immunity (Singh et al., 2022a; Cangui-Panchi et al., 2023). Phage therapy, recognized for its efficacy against antibiotic-resistant bacteria, is being explored by several research groups worldwide (Pires et al., 2020). Technological advancements have facilitated phage-based therapy research, revealing promising results in addressing antibiotic-resistant bacterial infections. Notably, phages have reduced contamination in medical catheters (Joo et al., 2023; Erol et al., 2024). However, phage application shows potential for the sanitation of medical instruments and holds great promise for a prospective application in clinical practice and patient treatment (Suh et al., 2022).

### 5.1 Environmental sanitation

#### 5.1.1 Phage application: prevention of biofilm in water environment

Contamination of water sources by enteric pathogens poses a significant threat to public health. These microorganisms can originate from various human activities, such as runoff from livestock facilities, intensive farming practices, and wastewater treatment plants (WWTPs) effluents. Infrastructure failures in sewage systems can exacerbate this issue, resulting in fecal pollution of surface waters (Shivaram et al., 2023). Traditional methods of microbial removal include chemical disinfection and antibiotic treatments, contributing to the emergence of antibiotic resistance among waterborne pathogens (Mancuso et al., 2021).

Bacteriophage therapy has emerged as a promising approach to combat antibiotic-resistant bacteria (Karthikeyan and Meyer, 2006; Shivaram et al., 2023). In WWTPs, filamentous bacteria commonly cause bulking and foaming, reducing treatment efficiency (Ballesté et al., 2022). Laboratory studies have shown that isolated phages targeting these foam-causing bacteria can prevent foam stabilization, offering potential solutions to this issue (Petrovski et al., 2011; Liu et al., 2015). Moreover, bacterial biofilms on membrane-based WWTPs can impair treatment processes (Wu et al., 2017). Lytic phages have demonstrated effectiveness in inhibiting bacterial fouling of membranes (Goldman et al., 2009; Bhattacharjee et al., 2015; Ayyaru et al., 2018); for instance, phage DTP1 showed promise as a biocontrol agent against *Delftia tsuruhatensis* ARB1, an antibiotic-resistant bacterium commonly found in WWTPs (Bhattacharjee et al., 2015).

*P. aeruginosa* biofilms frequently clog filters in water treatment plants and increase cleaning costs (Tian et al., 2021). Studies have explored the use of phages in combination with other disinfectants to achieve significant reductions (up to 96% removal rate) in *P. aeruginosa* biofilms, highlighting the potential of phage-based interventions in water treatment (Zhang and Hu, 2013). Antibiotic-resistant *Vibrio* species have been isolated from aquatic environments like dams, boreholes, and tap water (Maje et al., 2020); in aquaculture, preventive antibiotic treatment is commonly applied to reduce pathogenic *Vibrio* spp. and its biofilm (Tian et al., 2021; Lomelí-Ortega et al., 2023; Srisangthong et al., 2023). Also, lysins derived from a *Vibrio parahaemolyticus* phage have been proposed as a promising alternative to reducing antibiotic overuse in aquaculture (Matamp and Bhat, 2019). Overall, there is great potential in using phage-based interventions to eliminate waterborne pathogen contamination and reduce the reliance on antibiotics in water treatment and aquaculture practices.

#### 5.1.2 Phage application: prevention of biofilm in the food industry

In agriculture and livestock food production, infectious diseases pose significant challenges to food sustainability, causing crop losses, animal welfare issues, and environmental pollution with antimicrobial agents (Abebe et al., 2020). These diseases can also lead to emerging infectious diseases in humans and animals, facilitated by zoonotic transmission from microbial contamination (Rohr et al., 2019). Biofilms formed by foodborne pathogens have substantial public health implications, particularly in mixed-species biofilms common in the food industry (Galié et al., 2018). Bacteriophages have also been proposed as a green biocontrol tool for eliminating these microorganisms in food production (Moye et al., 2018).

Research has employed bacteriophages (Phage K and T4-like phages) for the treatment of *S. aureus* and *E. coli* that induced mastitis, a prevalent infectious disease in livestock (da Silva Duarte et al., 2018; Loponte et al., 2021). In 2006, the U.S. Food and Drug Administration (FDA) approved a phage cocktail sanitation product (ListShieldTM) to eliminate *Listeria monocytogenes* and marked a milestone for subsequent phage-based food preservation products (Zhang, 2018; Vikram et al., 2021). Biofilm formation on food production surfaces, often resistant to biocides, is a significant challenge (Meade et al., 2020). A recent study assessed the effectiveness of phage cocktails in the elimination of Shiga-toxigenic *E. coli* (STEC) growth over several types of surfaces (polystyrene well plates, stainless, steel, and high-density polyethylene) and found that STEC populations were reduced to undetectable levels after 16 hours of treatment (Jaroni et al., 2023). Similarly, another study used phage phT4A to reduce *E. coli* biofilm growth over plastic and reported its maximum inhibition percentage after only 6 hours of phage application (Brás et al., 2024).

Some studies have assessed the potential use of phage cocktails to combat dual-species biofilms, which are also relatively common biofilm arrangements over food production surfaces (Kim et al., 2023b). The effectiveness of these approaches depends on the combination of bacteria inside the mixed-species biofilm. For instance, one study showed that phages are more effective in eliminating single species than mixed biofilms in an *E. coli* and *Salmonella enteritidis* biofilm (Milho et al., 2019). In contrast, it has been suggested that phages tackling *Staphylococcus lentus* and *Pseudomonas fluorescens* mixed biofilms are able to eliminate their respective host within the biofilm (Sillankorva et al., 2010; Milho et al., 2019). Although studies have demonstrated the efficacy of phages in controlling single-species biofilms, further research is needed to explore their application in mixed-species biofilms in food environments.

### 5.2 Clinical treatment

**Figure 2:**
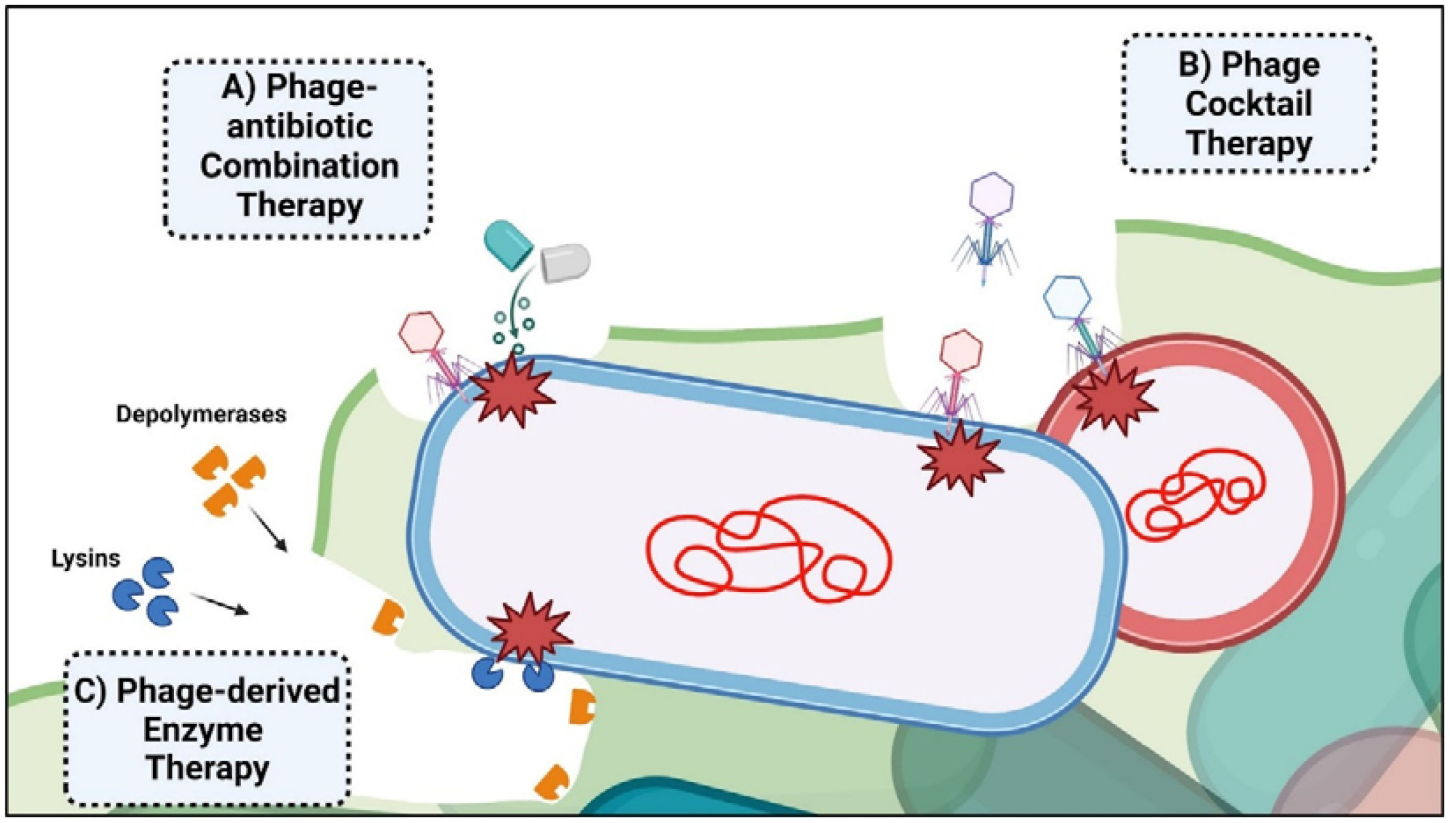
Bacteriophage-based therapeutic approaches employed for biofilm-derived infection. The figure illustrates the three main phage-associated mechanisms employed for clinical treatment. **(A)** Phage-antibiotic combination therapy can disrupt biofilm formation, making bacteria more vulnerable to phage attack and the effects of antibiotics. **(B)** Phage cocktail therapy involves using a mixture of bacteriophages to target and eliminate multiple strains or species of bacteria in poly-bacterial infections. **(C)** Phage-derived enzyme therapy utilizes enzymes, such as lysins and depolymerases, to degrade bacterial cell walls and biofilm matrices, degrading biofilms and eliminating bacteria.

The global impact of antibiotic-resistant bacteria, contributing to over 1.27 million annual deaths, underscores the urgent need for novel treatments (Antimicrobial Resistance Collaborators, 2022; Fongang et al., 2023). Bacterial biofilms are particularly problematic due to their ability to cause chronic and resilient infections. These biofilms offer protection and tolerance to antibiotics, antiseptics, antimicrobials, and host immune responses, leading to persistent infections (Yang et al., 2012). Eradicating biofilms within a host proves difficult due to physiological and physical barriers. Moreover, the minimum inhibitory concentration of antibiotics required against biofilms can be substantially higher than planktonic bacteria (Høiby et al., 2011). Biofilms on medical surfaces, such as catheters and implants, exacerbate the problem (Srivastava et al., 2019; Joo et al., 2023; Erol et al., 2024). Phage therapy has demonstrated significant potential in combating these infections through various therapeutic approaches (**Figure 2**). A summary of bacteriophage-based clinical studies is shown in **Table 1**.

**Table 1:** Clinical studies regarding bacteriophage therapy used to treat biofilm-related infections.

Target bacteria	Biofilm model	Treatment	Outcomes	Ref.
**Multiple species**	Chronic wound infection	Phage cocktail-antibiotic combination (ceftazidime-avibactam)	Infection was resolved in 90 days	(Karn et al., 2024)
***Pseudomonas aeruginosa***	Left Ventricular Assist Device Driveline Infection	Phage cocktail-antibiotic combination (ceftazidime/avibactam-amikacin)	Infection was resolved. No adverse effects were observed	(Racenis et al., 2023)
***Enterococcus faecalis***	Prosthetic joint infections	Phage-antibiotic combination (daptomycin and amoxicillin)	*E. faecalis* infection was resolved but the patient presented a posterior MRSA infection.	(Doub et al., 2023)
***Pseudomonas aeruginosa***	Prosthetic vascular graft infection	Phage cocktail-antibiotic combination (ceftazidime-avibactam)	Therapy did not resolve the infection	(Blasco et al., 2023)
***Pseudomonas aeruginosa***	Hip prosthetic infection	Phage-antibiotic combination (meropenem)	Infection was resolved. No adverse effects were observed	(Cesta et al., 2023)
***Staphylococcal* spp.**	Peri-Prosthetic joint infections	Phage cocktail-antibiotic combination	Lower rate of periprosthetic joint infection relapse	(Fedorov et al., 2023)
**MRSA**	Knee and hip prosthetic joint infection	Phage-antibiotic combination (daptomycin and vancomycin)	Infection was resolved. No adverse effects were observed	(Schoeffel et al., 2022)
**MRSA**	Prosthetic joint infection	Phage-antibiotic combination (daptomycin)	Infection was resolved. No adverse effects were observed	(Doub et al., 2022a)
***Klebsiella pneumoniae***	Prosthetic shoulder infection	Phage cocktail combination (ertapenem)	Infection was resolved. No adverse effects were observed	(Doub et al., 2022b)
***Pseudomonas aeruginosa***	Left Ventricular Assist Device Driveline Infection	Phage-antibiotic combination (Ceftolozane/tazobactam)	Infection was resolved. No adverse effects were observed	(Tkhilaishvili et al., 2021)
Staphylococcus aureus*	Pelvic bone allograft infection	Phage-antibiotic combination (intravenous clindamycin, rifampin and ciprofloxacin)	Therapy did not resolve the infection	(Van Nieuwenhuyse et al., 2021)
***Staphylococcus epidermidis***	Prosthetic knee infection	Phage-antibiotic combination (daptomycin and doxycycline)	Phage therapy discontinued by patient request due to mild transaminitis	(Doub et al., 2021)

MRSA, methicillin-resistant S. aureus.

(*Multispecies infection was diagnosed but only S. aureus phages were available for the phage therapy).

#### 5.2.1 Bacteriophage and antibiotic combination therapy

The use of bacteriophages and antibiotics during a combination therapy has shown a synergizing effect that increases the effectiveness of biofilm eradication. This approach reduces antibiotic concentrations while maintaining efficacy (Morrisette et al., 2020). In 2018, a study assessed the synergistic effect of pre-treatment application of Sb-1 *S. aureus* phage followed by antibiotic administration and demonstrated the effective elimination of MRSA biofilms (Tkhilaishvili et al., 2018). Previously, combination therapy of T4 phage and antibiotics showed potential to reduce biofilms formed by antibiotic-resistant and phage-resistant *E. coli* biofilms (Coulter et al., 2014). Notably, studies in *P. aeruginosa* biofilms using phage-antibiotic combination therapy also demonstrated high efficacy. In 2017, one study assessed the effects of the concurrent administration of a 12-phage cocktail PP1131 with various antibiotics. It concluded that this combination exhibited efficacy in treating bacterial infections in laboratory rats, compared to administering only one of the components individually (Oechslin et al., 2017).

More recently, some clinical studies have already applied the combination of phage and antibiotic treatments in patients with recurrent or unresolved infections (**Table 1**). For instance, during a case study, a 62-year-old woman was treated with a combination of phage Pa53 and meropenem to treat a chronic right hip prosthesis infection caused by *P. aeruginosa* since 2016. The patient could self-administer the phage suspension with no observable side effects during the treatment. A 2-year follow-up evaluation concluded the success of the treatment as no signs of infection relapse were found (Cesta et al., 2023). The question regarding the effectiveness of phage application with other types of drugs has also started to be investigated. One study observed that certain anticancer drugs can inhibit the activity of potential therapeutic phages in an *S. aureus* infection model (Li et al., 2023). Immunocompromised cancer patients are highly vulnerable to opportunistic infection; for example, lung cancer patients can present high rates of *S. aureus*-derived pneumonia during chemotherapy (Rolston, 2017). Chemotherapeutic drugs can be seen as a limitation for using antibiotic approaches such as antibiotics or phage therapy due to interference or incompatibility. Li and coworkers studied the synergetic potential of phages with common anti-cancer drugs. Combining phage K with Doxorubicin effectively eliminated *S. aureus* intracellular infections and migration (Li et al., 2023).

#### 5.2.2 Bacteriophage cocktail therapy

Recent findings suggest the efficacy of phage cocktails against bacterial biofilms, particularly in multispecies environments, offering advantages like a broader host range and reduced risk of phage resistance (Morrisette et al., 2020). Using multiple phages enables the recognition of different bacterial receptors, which increases their effectiveness (Singh et al., 2022b; Naknaen et al., 2023). For example, a study utilized six lytic phages against a wide range of *P. aeruginosa* clinical isolates and assessed biofilm-inhibiting in static and dynamic biofilm models. The phage cocktail effectively eliminated most biofilm biomass within 4 hours for the static model and within 48 hours in the dynamic biofilm model (Alves et al., 2016). Another promising phage cocktail (EFDG1 and EFLK1) was able to target and eliminate planktonic and biofilm cultures of vancomycin-resistant *E. faecalis* V583 strains (Khalifa et al., 2018).

A major pathogen in orthopedic and joint implant infections is *Staphylococcus*, specifically coagulase-negative *Staphylococcus* or MRSA (Bozhkova et al., 2013; Lu et al., 2022). Using phage cocktails combined with linezolid reduced adherence to MRSA after being surgically implanted in the intramedullary canal of the mouse femur bone (Kaur et al., 2016). Finally, in a phase I clinical trial, nine patients with *S. aureus*-derived chronic rhinosinusitis were treated with intranasal irrigants of phage cocktail AB-SA01 and presented no serious adverse effects (Ooi et al., 2019).

More recently, the application of phage cocktails and antibiotic courses has already been investigated in several clinical trials. For example, during a case study, a 54-year-old male patient was treated with a combination of two phages (PNM and PT07) and antibiotics (ceftazidime/avibactam and amikacin) to treat a left ventricular assist device driveline infection caused by multidrug-resistant *P. aeruginosa*. Follow-up evaluations were recorded at six weeks, 34 weeks, and 48 months after intervention, where the patient showed no signs of relapse (Racenis et al., 2023). The same year, a necessary clinical trial assessed the effect of an antibiotic phage cocktail in combination with several etiotropic antibiotics (Fedorov et al., 2023). During this trial, twenty-three adult patients with deep periprosthetic joint infection received phage-antibiotic therapeutic intervention during and after surgery, with two patients presenting mild adverse effects (fever) after phage administration, which were eventually resolved. In the end, the periprosthetic joint infection relapse rate was around 12 times lower than that in the control group (Fedorov et al., 2023).

#### 5.2.3 Bacteriophage-derived enzymes application

Several limitations can interfere with phage penetration, propagation, and diffusion through the EPS matrix, hindering the use of lytic phages to eliminate biofilms. Phage-derived enzymes, namely endolysins and depolymerizes, have emerged as potential mechanisms of EPS matrix degradation, as explained in Section 4.1. The efficacy of endolysins as tools for biofilm treatment has been extensively covered in the literature (de Miguel et al., 2020; Ghose and Euler, 2020; Abdelrahman et al., 2021).

In the field of clinical trials, some advancements have been made in the elimination of biofilm-forming bacteria of medical relevance. For instance, research has demonstrated that the phage depolymerase KPO1K1 can eliminate *Klebsiella pneumoniae* B5055 even on an old biofilm matrix (Fernandes and São-José, 2018). Recently, the multidrug-resistant clinical strain Kl 315 of *K. pneumoniae* and its biofilm were also shown to be effectively eliminated by phage displaying depolymerase activity (Zurabov et al., 2023).

In this regard, both endolysins and depolymerizes exhibit a narrow antibacterial spectrum, offering advantages over broad-spectrum antibiotics. They are suitable for selective elimination of drug-resistant pathogenic bacteria with lesser adverse effects on the host microbiome (Singh et al., 2022b).

#### 5.2.4 Novel bacteriophage-based clinical approaches

In recent years, novel bacteriophage approaches have gained significant attention as promising strategies for treating biofilm-associated infections, effectively addressing the limitations of traditional antibiotics and earlier phage therapies. These approaches have been assessed *in vivo* models and tackle critical challenges such as efficient phage delivery, resilience against harsh environmental conditions within the host, neutralization by host antibodies, and the internalization of phages to combat intracellular infections. As mentioned in Section 4, bacteriophage encapsulation represents an innovative method for biofilm infection treatment, leveraging bacteriophages’ inherent potential while overcoming issues related to their stability and targeted delivery (Yan et al., 2021).

Clinical applications of hydrogel-based bacteriophage encapsulation have shown significant potential *in vivo* for treating multidrug-resistant (MDR) infections. In 2015, a study evaluated the temperate phage ФPan70 for its efficacy against MDR *P. aeruginosa* in planktonic, biofilm, and mouse burn models. The phage significantly reduced bacterial populations and improved the survival rate of burned mice from 80% to 100%. Researchers suggested that phages could prevent bacterial spread into the bloodstream and enhance immune responses at the injection site (Holguín et al., 2015).

**Figure 3:**
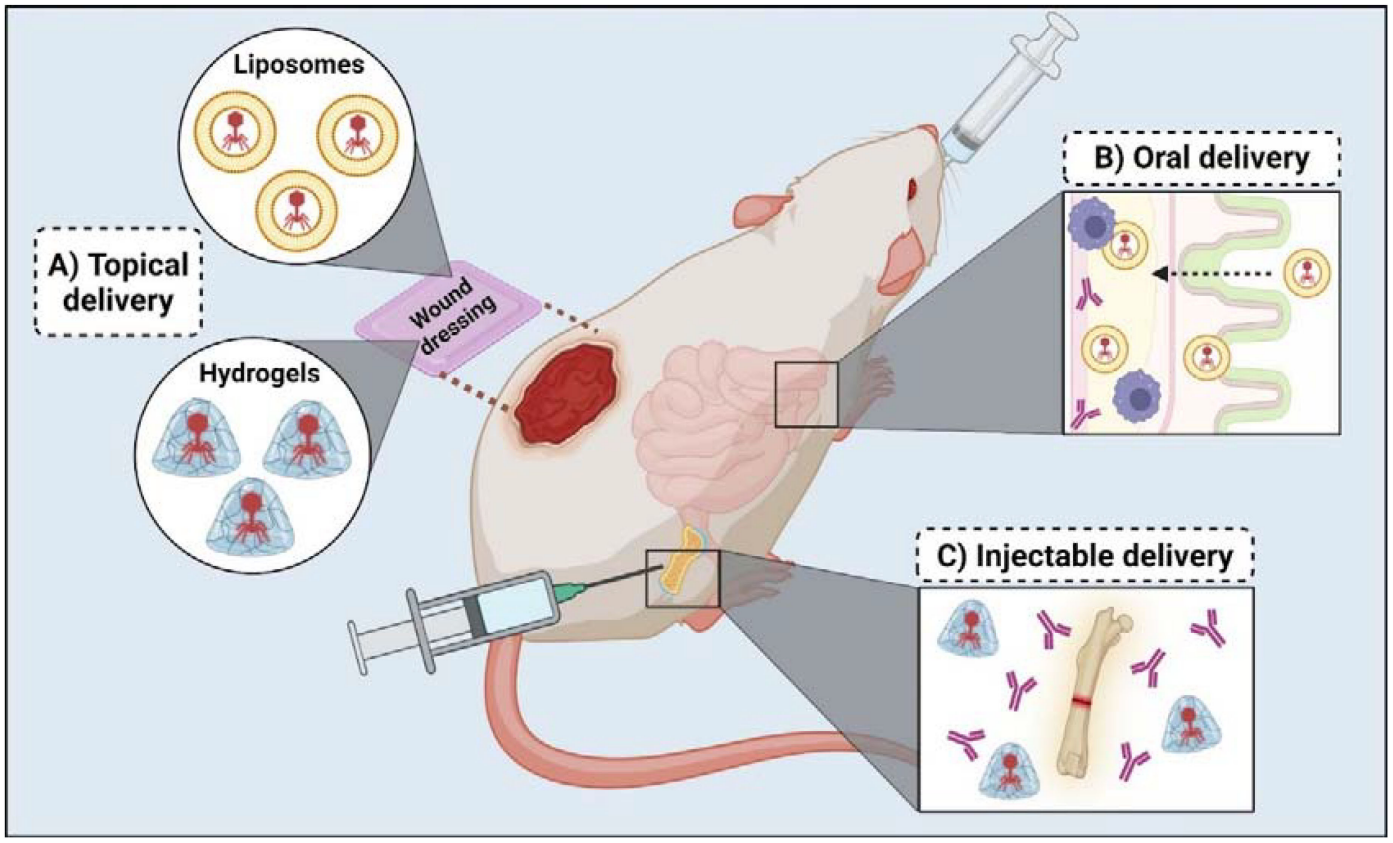
*In vivo* studies regarding bacteriophage delivery for clinical application. **(A)** Topical delivery has been used in mice wound models and involves wound dressing coated with liposomes or hydrogels containing bacteriophages. **(B)** Oral delivery mainly uses liposome encapsulation to protect bacteriophages on their transit to the intestinal tract, facilitate transportation through the intestinal layer and avoid antibody interference. **(C)** Injectable delivery allows the intradermal and intramuscular application of bacteriophage therapy in case of bone and prosthetic joint infection; it usually involves using hydrogels to protect bacteriophages from antibodies and other immune responses.

Further advancements in wound therapy have led to the development of wound dressings composed of chitosan, sodium alginate, and carboxymethyl cellulose, targeting MDR bacteria and biofilm-mediated infections in mice wound models (Mabrouk et al., 2022; Dehari et al., 2023; Mukhopadhyay et al., 2023). For instance, a study highlighted the synergistic effect of a phage-ciprofloxacin hydrogel in a mouse wound healing model, showing enhanced wound healing and improving mice recovery (Shafigh Kheljan et al., 2023). **Figure 3** showcases the concept of wound dressing with hydrogel-coated bacteriophages. Some studies suggest that hydrogel-coated bacteriophages can be delivered via injectable application into specific tissues. For instance, a recent study utilized a fracture-related infection (FRI) mouse model to assess the effectiveness of a hydrogel containing both a phage cocktail and antibiotic meropenem to treat a *P. aeruginosa* FRI. Compared with the application of free bacteriophages, the phage-meropenem hydrogel exhibited a lower incidence of phage resistance and reduced serum neutralization (Chen et al., 2023).

On the other hand, encapsulating bacteriophages in liposomes also shows promise in enhancing their stability and protection from the host immune system, ensuring controlled release at the infection site (**Figure 3**). Studies have employed various methods for effective phage encapsulation, such as a microfluidic-based technique to create liposomes with adjustable sizes (Cinquerrui et al., 2018). In a 2016 study, liposomes were evaluated for their ability to deliver phages intracellularly and their susceptibility to anti-phage antibodies. Liposome-encapsulated phages were fully protected from neutralizing antibodies, whereas free phages were neutralized within three hours. Additionally, liposome-encapsulated phages were able to enter mouse peritoneal macrophages and achieve a 94.6% killing rate of intracellular *K. pneumoniae* (Singla et al., 2016).

Another *in vivo* study in mice examined the biodistribution of orally administered, liposome-encapsulated fluorescent bacteriophages and their transport through intestinal cell layers. This study showed that liposome-encapsulated phages persisted longer in the stomach and adhered to the intestinal membrane, suggesting greater long-term efficacy of phage therapy (Otero et al., 2019). Murine models are not the only *in vivo* models used for these studies. For instance, in 2022, a study demonstrated that asymmetric phosphatidylserine/phosphatidic acid (PS/PA) liposomes, used in both prophylactic and treatments, reduced bacterial burden in zebrafish embryos infected with *P. aeruginosa* by enhancing macrophage phagocytic activity. In this regard, combining liposomes and phage cocktails improves antimicrobial effects and is effective against phage-resistant bacteria (Cafora et al., 2022).

Phage engineering has also shown great potential to develop novel approaches for targeting infections in clinical settings. Genetic modifications can enhance the expression of lytic enzymes, such as phage hydrolases, and facilitate the degradation of the biofilm matrix. For instance, genetically modified (GM) T7 *E. coli* phages have enhanced biofilm matrix degradation efficacy (Waturangi et al., 2021).

Studies have shown that phages can be engineered to augment their antimicrobial properties. In 2023, modification of the capsid of K1F phages increased their affinity for human tissue, potentially enhancing their ability to target intracellular infections caused by their host bacteria, *E. coli* K1 (Williams et al., 2023). Furthermore, eliminating lysogeny-related genes in Ef11 *E. faecalis* phages has shown promising results in eradicating *E. faecalis* biofilm populations, including vancomycin-resistant strains (Tinoco et al., 2016). Overall, genetic modification of phages also represents a valuable tool to enhance the effectiveness of phage-based biofilm treatment in clinical settings.

## 6 Future perspectives and challenges

Bacteriophage therapy, an innovative approach for targeting biofilm-associated infections and antibiotic-resistant bacteria, holds significant potential. However, its application is accompanied by significant challenges and limitations that necessitate critical examination. These challenges encompass a range of technical, biological, and regulatory issues that must be addressed to harness the full potential of phages in clinical and environmental applications.

### 6.1 Enhancing phage efficacy and delivery

A primary limitation of bacteriophage therapy is its narrow host range, which restricts its application to specific bacterial strains (Magaziner and Salmond, 2022; Ratnakar, 2022). This specificity, though beneficial in minimizing off-target effects, significantly limits the broader clinical applicability of phage therapy, especially in polymicrobial infections. Phage cocktails, comprising multiple phages with varying host ranges, have been suggested as a solution to this limitation (Schooley et al., 2017; Zaczek-Moczydłowska et al., 2020). However, the formulation of such cocktails must carefully consider the potential antagonistic interactions between phages, which could compromise their efficacy. Additionally, identifying and formulating effective phage cocktails is resource-intensive and requires extensive screening and validation.

### 6.2 Addressing phage resistance

Similar to antibiotics, bacteriophages face the challenge of bacterial resistance. Bacteria can evolve mechanisms to evade phage predation (Teklemariam et al., 2023), such as altering surface receptors required for phage attachment and infection (Rendueles et al., 2023). This phenomenon requires ongoing monitoring and the development of new phages, a process that demands significant resources and time. To mitigate resistance, strategies such as combining phages with antibiotics have shown promise in enhancing biofilm eradication and preventing resistance (Lu et al., 2023; Meneses et al., 2023).

### 6.3 Structural complexity of biofilms

The dense extracellular matrix of biofilms presents a significant barrier to effective phage therapy. This matrix can impede phage penetration and reduce bactericidal efficiency. Advanced delivery systems, including nanoparticles, liposomes, and hydrogels, are being explored to enhance phage stability and facilitate deeper penetration into biofilm structures (Kluzek et al., 2022; Costa et al., 2023). Despite promising results, these delivery mechanisms’ development and clinical application remain in their early stages and require further validation.

### 6.4 Stability in various environments

The stability of phages in different environments, including the human body, is a concern. Factors such as pH, temperature, and the presence of immune components can influence phage viability and activity. Strategies like encapsulating phages in protective carriers or engineering phages to evade immune detection are under investigation (Arias et al., 2022; Hufziger et al., 2022; Gao and Feng, 2023). However, these approaches add complexity to treatment protocols.

### 6.5 Environmental and industrial applications

Beyond clinical settings, phage therapy holds potential for environmental sanitation and industrial applications. Phages can be utilized to control biofilms in water treatment plants (Shivaram et al., 2023), food processing environments (Vikram et al., 2021; Brás et al., 2024), and agricultural settings (Ascaño et al., 2022), providing sustainable and effective alternatives to chemical treatments. Future research should focus on optimizing phage application methods to ensure environmental compatibility and effectiveness across diverse conditions.

### 6.6 Phage-host interactions and evolutionary dynamics

Understanding the complex interactions between phages and their bacterial hosts is crucial for successfully applying phage therapy. Phage-host dynamics are influenced by various factors, including the genetic diversity of phages and bacteria, environmental conditions (Fister et al., 2016; Goehlich et al., 2024), and the presence of other microorganisms (Alkhalil, 2023). Studying these interactions can provide insights into the mechanisms of bacterial resistance and inform the development of more effective phage therapies.

### 6.7 Integration with other therapies

Combining phage therapy with other treatment modalities, such as antibiotics, antimicrobial peptides, and immune modulators, has shown promise in enhancing therapeutic outcomes (Coulter et al., 2014; Morrisette et al., 2020; Joo et al., 2023; Lu et al., 2023). This approach requires careful consideration of potential interactions, dosing regimens, and treatment protocols. Clinical trials and studies are needed to evaluate the safety and efficacy of combination therapies and establish guidelines for their use in clinical practice.

### 6.8 Release of endotoxins by lysis of Gram-negative bacteria

A critical impediment to the deployment of phage therapy for infections caused by Gram-negative bacteria is the simultaneous release of endotoxins, particularly LPS, during the lysis of bacterial cells. Gram-negative bacteria are characterized by an outer membrane enriched with LPS, a potent elicitor of human immune responses. Bacteriophage-induced lysis releases these cell wall components, including endotoxins, into the extracellular milieu, precipitating a severe inflammatory reaction. This response, known as endotoxin shock or septic shock, is marked by symptoms such as fever and hypotension, which can lead to fatal outcomes if not adequately controlled. The efficacy of phage therapy in reducing bacterial populations is paradoxically undermined by the exacerbation of clinical symptoms due to the rapid liberation of endotoxins (Hibstu et al., 2022). Strategies to mitigate this issue include the development of genetically engineered phages designed to either preclude the release of endotoxins or facilitate their degradation post-release (Matsuda et al., 2005; Łobocka et al., 2021).

In conclusion, while bacteriophage therapy offers a promising alternative to traditional antibiotics, continued research, innovation, and a multidisciplinary approach that addresses its myriad challenges and complexities is needed. The path forward involves enhancing phage delivery systems, developing strategies to mitigate resistance, expanding host range specificity, navigating the regulatory landscape, and fostering public engagement to ensure phage therapy’s sustainable and effective application across multiple domains.
